# Relationship between Testicular Volume and Conventional or Nonconventional Sperm Parameters

**DOI:** 10.1155/2013/145792

**Published:** 2013-09-05

**Authors:** Rosita Condorelli, Aldo E. Calogero, Sandro La Vignera

**Affiliations:** Section of Endocrinology, Andrology and Internal Medicine, Department of Medical and Pediatric Sciences, University of Catania, Policlinico “G. Rodolico”, Via S. Sofia 78, Building 4, Room 2C19, 95123 Catania, Italy

## Abstract

*Background*. Reduced testicular volume (TV) (<12 cm^3^) is associated with lower testicular function. Several studies explored the conventional sperm parameters (concentration, motility, and morphology) and the endocrine function (gonadotropins and testosterone serum concentrations) in the patients with reduction of TV. No other parameters have been examined. *Aim*. This study aims at evaluating some biofunctional sperm parameters by flow cytometry in the semen of men with reduced TV compared with that of subjects with normal TV. *Methods*. 78 patients without primary scrotal disease were submitted to ultrasound evaluation of the testis. They were divided into two groups according to testicular volume: A Group, including 40 patients with normal testicular volume (TV > 15 cm^3^) and B Group, including 38 patients with reduced testicular volume (TV ≤ 12 cm^3^). All patients underwent serum hormone concentration, conventional and biofunctional (flow cytometry) sperm parameters evaluation. *Results*. With regard to biofunctional sperm parameters, all values (mitochondrial membrane potential, phosphatidylserine externalization, chromatin compactness, and DNA fragmentation) were strongly negatively correlated with testicular volume (*P* < 0.0001). *Conclusions*. This study for the first time in the literature states that the biofunctional sperm parameters worsen and with near linear correlation, with decreasing testicular volume.

## 1. Introduction

 The testis is an oval-shaped organ that in adult men weighs about 20 g which has an average volume (TV) of 18.6 ± 4.8 cm^3^ and dimensions ranging from 3.6 to 5.5 cm in length and from 2.1 to 3.5 cm in width. Testis consists of two structurally distinct compartments: the interstitial compartment and the seminiferous tubules containing germ and Sertoli cells. These two compartments are responsible for the two main testicular functions: production of androgens and spermatozoa, respectively [1]. About 400–600 seminiferous tubules are found within the testis; they are 70–80 cm long, have a diameter of 0.12-0.13 mm, and represent about 80–90% of the total TV. 

 In clinical practice, the TV can be evaluated by physical examination using the Prader's orchidometer, but with a greater precision by testicular ultrasound which overcomes confounding factors such as scrotal effusions, varicose veins, intrascrotal masses, testicular pain, and distinction from adjacent anatomical structures [2]. It is well known that a significant TV decrease is associated with a worst testicular performance of both endocrine (androgen decline) and reproductive activities (abnormal sperm parameters) [3–6]. A number of studies have explored the relationship between conventional sperm parameters (concentration, motility, and morphology) or the endocrine function (testosterone and gonadotropin serum concentrations) and TV [7–9]. No other parameters have been taken into account. Therefore, the aim of this study was to evaluate nonconventional sperm parameters (mitochondrial function, apoptosis, and chromatin/DNA integrity) in men with reduced TV. These parameters were compared with those evaluated in men with normal TV. 

## 2. Materials and Methods

### 2.1. Patient Selection

 We retrospectively reviewed the clinical, and laboratory (hormonal, conventional and nonconventional flow cytometry sperm parameters) charts of men who requested didymo-epididymal ultrasound scan for any reason from December 2011 to November 2012. Patients having a condition known to interfere with sperm parameters were excluded from analysis. These included the following:

- primary scrotal disease identified by ultrasound evaluation: varicocele, hydrocele, testicular focal injury/injuries, microlithiasis, inhomogeneous testicular echotexture, and abnormal testicular arterial hemodynamic (systolic peak velocity and resistance index);
- history of cryptorchidism, varicocelectomy, eversion of tunica vaginalis, head injury, endocrine abnormalities (hypogonadism, hyperprolactinemia, and increased estrogen concentration), systemic diseases (kidney disease, liver disease [10], and diabetes mellitus [11]), cigarette smoking [12], alcohol use [13], concomitant use of other drugs during the previous 6 months, leukocytospermia, male accessory gland inflammatory disease [14], and positive seminal and/or urine culture and/or urethral swab.

Applying these exclusion criteria, 78 patients were included in this study. They had a mean age of 25.0 ± 5.0 years (range 23–45 years) and an average BMI of 25.0 ± 3.0 kg/m^2^ (range 20–30 kg/m^2^). The testicular size was considered normal when the mean TV ranged between 15 mL and 25 mL [15, 16], whereas it was considered low when TV was <12 mL. Patients with a mean TV in a grey zone (12–14.9 mL) were arbitrarily excluded from computation [6, 17, 18].

### 2.2. Scrotal Ultrasound Evaluation

 Scrotal ultrasound scans were carried out in two phases: the first with the patient in supine position (with penis resting on suprapubic region) and the second in upright position to evaluate blood reflux along the pampiniform plexus, testicular malposition, or extent of any fluid collections. Examination was conducted with a GX Megas Esaote (Esaote SpA, Genoa, Italy) instrument equipped with linear, high-resolution, and high-frequency (7.5–14 MHz) probe dedicated to the study of soft parts and with color Doppler for detecting slow flows and scanning surface of at least 5 cm.

 The TV was calculated automatically by the ultrasonography software by applying the ellipsoid formula (length × width × thickness × 0.52). Parenchymal echostructure was considered normal in presence of thin, densely packed and homogeneously deployed echoes. Presence of finely inhomogeneous echopattern, weakly hypo- or hyperechogenic areas was considered expression of primary testicular disease.

 During Doppler evaluation, flow was detected at the level of spermatic artery, testicular artery and its branches. Velocitogram was considered normal in presence of low resistance, prolonged systolic phase, flow maintained during the entire diastole, and low resistance index (IR: 0.62). 

### 2.3. Hormonal Measurements

Hormonal evaluation was performed by electrochemiluminescence with Hitachi-Roche equipment (Cobas 6000, Roche Diagnostics, Indianapolis, IN, USA). Blood sampling was performed at 8.00 am, after at least 8 hours of sleep. Determination of serum LH and prolactin was repeated at a distance of 30 minutes. Normal values were LH = 1.6–9.0 mIU mL^−1^, FSH = 2.0–12.0 mIU mL^−1^, total testosterone = 2.8–8.0 ng mL^−1^, 17*β*-estradiol = 8.0–43.0 pg mL^−1^, and prolactin = 4.0–15.0 ng mL^−1^.

### 2.4. Sperm Analysis

 Semen samples were collected by masturbation in a sterile container after 2–7 days of sexual abstinence and were transported to the laboratory within 30 minutes from ejaculation. Sperm analysis was made according to the WHO criteria [19]. The following parameters were evaluated: color, volume, liquefaction time, pH, density, total count, progressive motility, morphology, and leukocytes.

### 2.5. Evaluation of Nonconventional Sperm Parameters by Flow Cytometry

Analysis was performed using an EPICS XL Flow Cytometer (Coulter Electronics, IL, Italy), equipped with an argon laser at 488 nm and three detectors of fluorescence: green (FL-1 at 525 nm), orange (FL-2 to 575 nm) and red (FL-3 at 620 nm). For each sample, 100,000 events were counted at a low flow velocity and analysed by the software System II, Version 3.0. The following nonconventional sperm parameters were evaluated: (a) sperm mitochondrial membrane potential (MMP), (b) phosphatidylserine externalization (PS) (early apoptosis index) following staining with annexin V and PI, (c) degree of chromatin compactness after staining with propidium iodide (PI), and (d) sperm DNA fragmentation by TUNEL assay.

#### 2.5.1. Mitochondrial Membrane Potential Evaluation

 An aliquot containing 1 × 10^6^ spermatozoa was incubated with JC-1 (5,5′,6,6′-tetrachloro-1,1′,3,3′-tetraethylbenzimidazolylcarbocyanine iodide) (Space Import Export, Milan, Italy) in the dark for 10 minutes at a temperature of 37°C. At the end of the incubation period, cells were washed in PBS and analysed.

#### 2.5.2. Phosphatidylserine Externalization

 Staining with annexin V and PI was performed using Annexin V-FITC kit (Sigma Chemical, Perth, Australia). An aliquot containing 0.5 × 10^6^ spermatozoa was suspended in 0.5 mL of buffer containing 10 *μ*L of annexin V-FITC plus 20 *μ*L of PI and incubated for 10 min in the dark. After incubation, the samples were immediately analysed. Signals were detected by FL-1 (FITC) and FL-3 (PI) detectors. Cell populations were identified in a plot forward (FSC) versus side scatter (SSC). Different staining patterns enabled the identification of different cell populations: FITC negative and PI negative indicated vital cells, FITC positive and PI negative indicated apoptotic cells, and FITC positive and PI positive indicated cells in late apoptosis phase or necrosis.

#### 2.5.3. Evaluation of Chromatin Compactness

 PI staining was performed after cell membrane permeabilisation to allow fluorochrome penetration at the nuclear level. Briefly, 1 × 10^6^ spermatozoa were incubated with LPR DNA-Prep Reagent containing 0.1% cyanide potassium, 0.1% NaN_3_, nonionic detergents, salts, and stabilizers (Beckman Coulter, IL, Milan, Italy), in the dark, at room temperature for 10 minutes and then incubated with Stein DNA-Prep Reagent containing 50 *μ*g/mL of PI (<0.5%), A RNAse (4 KUnitz/mL), <0.1% NaN_3_ (Beckman Coulter, IL, Milan, Italy) in the dark at room temperature. Flow cytometric analysis was performed after 30 minutes. In this analysis, FL3 detector was the only used.

#### 2.5.4. DNA Fragmentation Analysis

 Sperm DNA fragmentation was evaluated by TUNEL assay using a commercially available kit (Mebstain Apoptosis, Beckman Coulter, IL, Milan, Italy). To obtain the negative control, TdT was omitted from the mixing reaction. Positive control was obtained by pretreating spermatozoa with 1 mg/mL of I deoxyribonuclease not containing A RNAse at 37°C for 60 min before staining. Debris was removed using the same procedure. Light scattering and fluorescence data were obtained at a fixed setting in logarithmic scale. FITC-labeled spermatozoa were measured using the flow cytometer FL-1 detector.

The protocol was approved by the Institutional Review Board, and an informed written consent was obtained from each patient and controls.

### 2.6. Statistical Analysis

 Results were reported as mean ± SEM. The data were analysed by Student *t*-test. The Pearson correlation coefficient was used to assess the possible covariance linearity between TV and the laboratory parameters evaluated. Statistical analysis was performed using SPSS 9.0 for Windows (SPSS Inc., Chicago, IL, USA). A *P* value lower than 0.05 was accepted as statistically significant.

## 3. Results

 Altogether the patients had a mean TV of 15.2 ± 1.7 mL. Forty patients (51.3%) had a normal TV, whereas the remaining 38 (48.7%) had low TV. The conventional sperm parameters of the patients enrolled in this study are shown in Table 1. The pH of the seminal fluid did not vary significantly in patients with low TV compared to those with normal TV. These patients with low TV had a statistically significant lower seminal fluid volume, sperm concentration, and percentage of progressive motility and spermatozoa with normal form compared with men with normal TV (*P* < 0.05, Student's *t*-test). Finally, the percentage of immature germ cells was significantly higher in patients with low TV compared to patients with normal TV (*P* < 0.05, Student's *t*-test). The hormonal serum concentrations are reported in Table 2. LH, FSH, and prolactin levels were similar between patients with normal or low TV. These latter had significantly higher 17*β*-oestradiol levels and lower total testosterone concentration compared to patients with normal TV (Student's *t*-test). 

**Figure 1:**
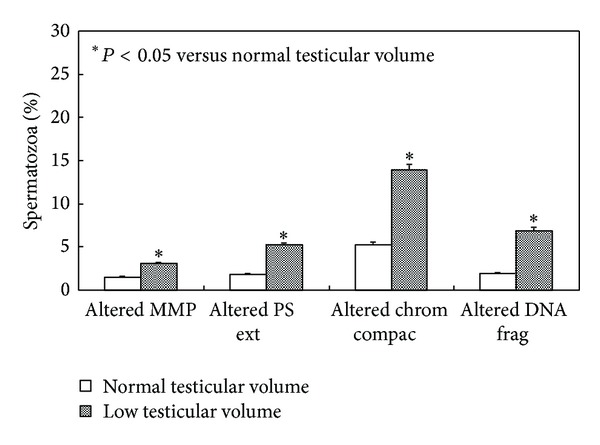
Percentage of spermatozoa with low mitochondrial membrane potential (MMP), phosphatidylserine externalization (PS ext), degree of chromatin compactness (chrom compac), or DNA fragmentation (DNA frag) in patients with normal (*n* = 40) or low (*n* = 38) testicular volume.

 Nonconventional sperm parameters are reported in Figure 1. The percentage of spermatozoa with low MMP, abnormal chromatin compactness, PS externalization, and fragmented DNA was significantly higher in patients with low TV compared to patients with normal TV (*P* < 0.05, Student's test).

**Figure 2:**
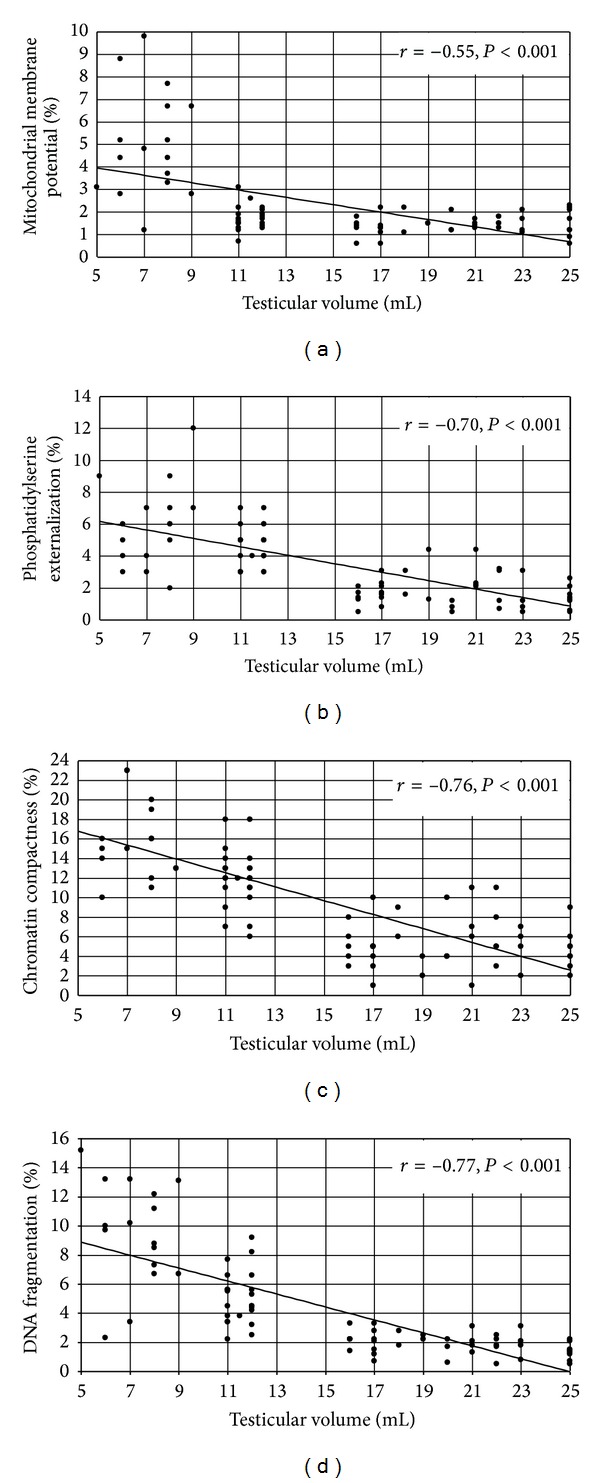
Correlation between mean testicular volume and percentage of spermatozoa with low mitochondrial membrane potential (a), phosphatidylserine externalization (b), degree of chromatin compactness (c), or DNA fragmentation (d) (*n* = 78).

The correlation analysis showed a significant direct correlation between TV and seminal volume, sperm concentration, progressive motility, and percentage of normal forms (Table 3). Statistically significant negative correlations were observed between TV and the percentage of immature germ cells and gonadotropin or 17*β*-oestradiol serum concentrations (Table 3). No correlation was found between TV and seminal pH, total testosterone, or prolactin serum concentration (Table 3). A statistically significant negative correlation was found between TV and low MMP, phosphatidylserine externalization, chromatin compactness, or DNA fragmentation (Figure 2).

## 4. Discussion

 This study explored the relationship between TV and conventional sperm parameters, serum hormonal concentrations, and using flow cytometry, nonconventional sperm parameters. To better understand the impact of TV on these parameters, the patients were selected excluding all cases of scrotal disease detected by ultrasound and excluding patients in whom the clinical history brought out factors that, directly (cryptorchidism, previous surgical trauma in the scrotal region, male accessory glands inflammatory disease, leukocytospermia, positive seminal, and/or urine culture and/or urethral swab) and/or indirectly (head trauma, systemic diseases, drugs, cigarette smoking, and alcohol use) affect testicular function and/or laboratory signs (endocrine diseases) that reflect testicular functional alterations. We found that patients with reduced TV have lower seminal fluid volume, sperm concentration, percentage of progressive motility, and percentage of spermatozoa with normal form, compared to men with normal TV. Conversely, these patients have an increased percentage of immature germ cells. These data suggest that patients with reduced TV (<12 cm^3^) show, in agreement with other observations [5, 6, 20], poorer sperm quality. It is noteworthy that these patients showed signs of tubular stress pattern as suggested by the increased percentage of immature germ elements in their ejaculate. 

In terms of hormonal concentrations, we found that patients with low TV have increased 17*β*-estradiol serum levels and lower concentrations of total testosterone. No statistically significant differences were detected in the concentrations of gonadotropins and prolactin between patients with normal, and low TV. These findings emphasize that a TV reduction may be a result of spermatogenic or hormonal impairment [21, 22], and not only on the quality of semen [5, 6, 20]. It is known that the endocrine testicular function has important implications on metabolism [23], cardiovascular profile [24], and bone metabolism [25]. In presence of low androgen concentrations peripheral insulin sensitivity is reduced [26], blood vessels are more predisposed to atherosclerosis [27] and, finally, osteoporosis is more common in patients with impaired testicular function. Recent findings have shown that the Leydig cell plays an important role in vitamin D metabolism [28].

At last, to our knowledge for the first time, this study investigated the relationship between TV and nonconventional sperm parameters. We found that patients with low TV have a lower number of spermatozoa with low mitochondrial membrane potential, early signs of sperm apoptosis (PS externalization), and an abnormal sperm chromatin/DNA integrity.

Only one similar study was carried out by Zorn and colleagues; in particular the authors evaluated phospholipid asymmetry, mitochondrial membrane potential, and DNA denaturation in 142 males of infertile couples showing that DNA denaturation correlated negatively with TV, normal mitochondrial membrane potential correlated positively with TV, and finally normal viable sperm correlated positively with TV [29]. However, the study does not examine the ultrasound appearance of the testis and does not identify a clear thresholds for TV estimated by ultrasound. 

The dilemma on the causes responsible for TV reduction, apparently unjustified on a clinical ground, remains open because of the absence of testicular disease identified by scrotal ultrasonography. In this regard, it is worth mentioning that some alterations of testicular morphostructural characteristics may be undetectable by physical and/or instrumental examination. In particular, chronic orchitis is difficult to clinically diagnose because of its asymptomatic course; however, the histological examination of many infertile patients detects lymphocytic peritubular infiltrate of inflammatory meaning in absence of relevant scrotal pathology, associated with TV and sperm count reduction [30, 31]. 

 In conclusion, the results of this study indicate that patients with a reduced TV (<12 mL), in the absence of testicular disease, show poorer conventional and nonconventional sperm parameters. The patients enrolled in this study did not show any frank endocrine alteration, but total testosterone serum concentration was lower in men with reduced TV. This suggests that men with low TV need to be monitored overtime for possible onset of an overt endocrine dysfunction. Finally, further studies are needed to clarify the relationship between TV reduction volume and ultrastructural alterations of the testicular parenchyma in patients with low TV and without evidence of disease at the clinical and ultrasound examinations. 

## Figures and Tables

**Table 1:** Conventional sperm parameters of men with normal (*n* = 40) or low (*n* = 38) testicular volume.

Parameter	Testicular volume	
	Normal (15–25 mL)	Low (<12 mL)
Volume (mL)	3.0 ± 0.5	2.3 ± 0.4*
pH	7.6 ± 1.2	7.7 ± 1.3
Sperm concentration (million/mL)	42.3 ± 6.7	28.7 ± 4.7*
Progressive motility (%)	36.8 ± 5.8	26.9 ± 4.4*
Normal forms (%)	13.0 ± 2.1	5.4 ± 0.9*
Immature germ cells (%)	3.5 ± 0.6	9.6 ± 1.6*

*P < 0.05 (Student's *t*-test).

**Table 2:** Serum hormone concentrations in men with normal (*n* = 40) or low (*n* = 38) testicular volume.

Parameter	Testicular volume	
	Normal (15.0–25.0 mL)	Low (<12.0 mL)
LH (mUI mL^−1^)	4.8 ± 0.8	5.0 ± 0.8
FSH (mUI mL^−1^)	4.4 ± 0.7	5.1 ± 0.8
Total testosterone (ng mL^−1^)	6.1 ± 1.0	5.2 ± 0.8*
17*β*-oestradiol (pg mL^−1^)	25.6 ± 4.1	35.8 ± 5.8*
Prolactin (ng mL^−1^)	16.9 ± 2.7	17.5 ± 2.8

*P < 0.05 (Student's *t*-test).

**Table 3:** Correlation between testicular volume and conventional sperm parameters or serum hormone levels (*n* = 78).

Parameter	*r*	*P* value
Conventional sperm parameters		
Seminal fluid volume (mL)	*r* = 0.47	<0.05
Seminal fluid pH	*r* = −0.11	NS
Sperm concentration (million/mL)	*r* = −0.42	<0.05
Spermatozoa with progressive motility (%)	*r* = 0.43	<0.05
Spermatozoa with normal morphology (%)	*r* = 0.82	<0.05
Immature germ cells (%)	*r* = −0.64	<0.05
Serum hormone concentrations		
Serum LH (mUI mL^−1^)	*r* = −0.31	NS
Serum FSH (mUI mL^−1^)	*r* = −0.37	NS
Serum total testosterone (ng mL^−1^)	*r* = 0.28	<0.05
Serum oestradiol (pg mL^−1^)	*r* = −0.35	<0.05
Serum prolactin (ng mL^−1^)	*r* = 0.02	NS

NS: not significant.
